# Lentinan -triggered butyrate-producing bacteria drive the expulsion of the intestinal helminth *Trichinella spiralis* in mice

**DOI:** 10.3389/fimmu.2022.926765

**Published:** 2022-07-28

**Authors:** Xuemin Jin, Yi Liu, Isabelle Vallee, Gregory Karadjian, Mingyuan Liu, Xiaolei Liu

**Affiliations:** ^1^State Key Laboratory for Zoonotic Diseases, Key Laboratory for Zoonosis Research, Ministry of Education, Institute of Zoonosis, College of Veterinary Medicine, Jilin University, Changchun, China; ^2^UMR BIPAR, Anses, Ecole Nationale Vétérinaire d’Alfort, INRA, University Paris-Est, Animal Health Laboratory, Maisons-Alfort, France; ^3^Jiangsu Co-innovation Center for Prevention and Control of Important Animal Infectious Diseases and Zoonoses, Yangzhou, China

**Keywords:** *Trichinella spiralis*, lentinan, mucin, gut microbiota, butyrate

## Abstract

Trichinellosis caused by *Trichinella spiralis* is a serious zoonosis with a worldwide distribution. Lentinan (LNT) is known to modulate the intestinal environment with noted health benefits, yet the effect of LNT against intestinal helminth is unknown. In our study, we first observed that LNT could trigger worm expulsion by promoting mucus layer functions through alteration of gut microbiota. LNT restored the abundance of *Bacteroidetes* and *Proteobacteria* altered by *T. spiralis* infection to the control group level. Interestingly, LNT triggered the production of butyrate. Then, we determined the deworming capacity of probiotics (butyrate-producing bacteria) in mice. Collectively, these findings indicated that LNT could modulate intestinal dysbiosis by *T. spiralis*, drive the expulsion of intestinal helminth and provided an easily implementable strategy to improve the host defence against *T. spiralis* infection.

## Introduction

Helminths are a major global health problem. Trichinellosis, caused by *Trichinella spiralis*, brings huge morbidities to the host population (1). *T. spiralis* infects wild and domestic animals through contaminated pig meat, as the major source for *Trichinella* transmission, which has a negative impact on the pork meat market. Prevention of this disease by interrupting helminth transmission includes drug and vaccine development for livestock. In the early stages of infection, the host expels *T. spiralis* from the intestine through ‘weep’, leading to hyperplasia of goblet cells and increased production of mucin (the ‘weep’) (2), suggesting that priming of mucus production could have evolved to protect against helminth infection.

The mucus layer overlies the intestinal epithelium, known as the first barrier. Thickening of the mucus layer is a common feature of intestinal helminth infection (3). One significant feature of intestinal homeostasis is the host microbiota, which plays a critical role in supporting pathogen defense (2, 4) and may actually be necessary for host defence against parasites (5, 6). Interestingly, the presence of specific bacteria in the gut shapes the glycan profile of mucus (7), which mediates protection against pathogens (8). The potential value of microbiota in the development of new therapies for helminth infection has attracted much attention (9). We therefore hypothesized that changes in gut microbiota influence the ability of the host to expel helminth through mucus.

β-glucan from barley could trigger worm expulsion dependent on the gut microbiota (10). However, β-glucan from different sources may have different functions. Lentinan (LNT), another β-glucan, is extracted from mushroom (11). Previously, we showed the adjuvanticity of LNT against helminth *T. spiralis* infection (12). It has been reported that oral administration of polysaccharides such as LNT can be utilized by intestinal microbiota, thereby contributing to microbiota changes (13). Supplementation with LNT orally increased the expression levels of mucin and attenuated virus –induced intestinal damage in the host through intestinal microbiota and their metabolites (14). However, the effect of LNT against intestinal helminth infection is not completely understood and the role of LNT -induced gut microbiota remains elusive.

In the present study, we assessed the efficacy of LNT on helminth *Trichinella* infection in the intestine. We found that LNT could trigger worm expulsion by promoting mucus layer functions. Moreover, the deworming capacity of LNT was examined along with the effect on gut microbiota composition. And LNT failed to reduce the helminth burden in mice with gut microbiota-dysbiosis due to impairment of the mucus layer, suggesting that gut mircobiota composition contributed to LNT -triggered worm expulsion. The specific beneficial bacteria were analysed and the related metabolites were used to expel *T. spiralis* in the intestine.

## Materials and methods

### Ethics statement

C57BL/6J mice (female, 4-6 weeks old) were purchased from the Norman Bethune University of Medical Science (NBUMS), China. Female Wistar rats were purchased from the Experimental Animal Centre of College of Basic Medical Sciences, Jilin University (Changchun, China) and kept in a temperature-controlled room (22 ± 2°C) under a 12 h dark–light cycle. All animal experiments were performed according to regulations of the Administration of Affairs Concerning Experimental Animals in China. The protocol was approved by the Institutional Animal Care and Use Committee of Jilin University.

### Helminths

The *T. spiralis* isolate (ISS534) was obtained from a naturally infected domestic pig in Henan Province in China. Briefly, Wistar rats were orally infected with 3000 infective larvae, and *T. spiralis* muscle larvae (ML) were recovered at 35 days post infection (dpi) (15).

### Lentinan administration experiment

To evaluate the role of type 2 immunity in the effect of LNT (MedChemExpress, HY-N6653) on *T. spiralis* infection, the mice were randomly divided into 5 groups (n =6 per group): (1) the control (Con) group were untreated, (2) the group were infected with 500 ML, (3) the group were administrated orally with LNT (100 mg/kg) daily from -14 dpi to 7 dpi and infected with 500 ML, (4) the group were administrated with STAT6 inhibitor, AS1517499 (20 mg/kg i.p) at 1, 3 and 5 dpi and infected with 500 ML, (5) the group were administrated orally with LNT (100 mg/kg) daily from -14 dpi to 7 dpi, AS1517499 (20 mg/kg i.p) at 1, 3 and 5 dpi and infected with 500 ML.

To verify the the role of microbiota in the effect of LNT on *T. spiralis* infection, the mice were randomly divided into 5 groups (n =6 per group): (1) the control (Con) group were untreated, (2) the group were infected with 500 ML, (3) the group were administrated orally with antibiotics (ampicillin 1 g/L, vancomycin 0.25 g/L, neomycin 1 g/L, and metronidazole 1 g/L) daily 2 weeks before *T. spiralis* infection (500 ML), (4) the group were administrated orally with LNT (100 mg/kg) daily from -14 dpi to 7 dpi and infected with 500 ML, (5) the group were run in parallel with antibiotics and LNT daily 2 weeks before *T. spiralis* infection (500 ML) and LNT administration continued for 1 week after infection. The mice were sacrificed using CO_2_ asphyxiation at 7 dpi and 35 dpi for collecting helminthes (intestinal adult worms and muscle larvae). Small intestine and content were immediately collected for the following experiment.

### Measurements of the small intestine mucus layers

Post Carnoy’s fixation, the methanol-stored duodenum samples were embedded in paraffin, cut into thin sections (5 μm), and deposited on glass slides. Periodic acid Schiff (PAS) staining was performed according to standard protocols. Quantification of the goblet cell number was carried out by blinded operators. The sections were incubated with anti- mucin 2 (Muc2) rabbit polyclonal antibody (1:400 dilutions; Abcam) for immunohistochemistry according to kit manufacturer instructions. PAS and immunohistochemistry of Muc2 in duodenum. Scale bars: 50 μm.

### Real time PCR

mRNA expression of mucin 2 (Muc2) were quantified using RT-PCR using SYBR Green QPCR Master Mix (TaKaRa, Japan) with an Applied Bioscience 7500 thermocycler and FastStart Universal SYBR Green Master (Roche Applied Science, Germany) as described previously (16). Briefly, RNA extraction was performed by lysing 100 mg of small intestine tissue samples with Trizol reagent (Invitrogen). The primers of Muc2 were as follow: 5′- CCTTAGCCAAGGGCTCGGAA-3′ and 5′- GGCCCGAGAGTAGACCTTGG-3′ (17). The relative mRNA expression levels of the target genes were normalized to those of the indicated housekeeping gene (GAPDH) (5′- ACTCCACTCACGGCAAATTC-3′ and 5′- TCTCCATGGTGGTGAAGACA-3) and were quantified using the comparative Ct method and the formula 2^-ΔΔCT^.

### Microbial analysis in small intestine contents

Genomic DNA amplification and sequencing were conducted. PCR amplification was performed as described previously (18). Briefly, microbial DNA was extracted from 100 mg of small intestine contents of mice under strict aseptic operation. Then DNA was stored at -80°C until further processing. The V3-V4 region of the bacterial 16S rRNA gene was amplified with the common primer pair (Forward primer, 5’- ACTCCTACGGGAGGCAGCA-3’; reverse primer, 5’- GGACTACHVGGGTWTCTAAT-3’) combined with adapter sequences and barcode sequences. Microbiome sequencing data have been deposited at the National Center for Biotechnology Information, Sequence Read Archive (PRJNA723732).

The bioinformatics analysis of this study was performed with the aid of the BMK Cloud (Biomarker Technologies Co., Ltd., Beijing, China). We processed the raw data with Trimmomatic (v0.33) to trim low quality reads. Cutadapt was used to discard forward and reverse primers. The paired-end reads that were obtained were merged using FLASH v1.2.11 software, and reads with a length ≥400 pb were kept for the following analysis. The UCHIME algorithm (v8.1) was used in detecting and removing chimera sequences to obtain the clean tags. Sequences with similarity ≥97% were clustered into the same operational taxonomic unit (OTU) by USEARCH (v10.0) (19), and the OTUs with reabundace <0.005% were filtered. Taxonomy was assigned to all OTUs by searching against the SILVA databases using the RDP classifier within QIIME2. The Alpha diversity and Beta diversity were calculated and displayed by the QIIME and R software, respectively. Furthermore, we employed Linear Discriminant Analysis (LDA) effect size (LEfSe). Based on the Kyoto Encyclopedia of Genes and Genomes (KEGG) functional pathway, the functional composition was prediceted for each sample using Phylogenetic Investigation of Communities by Reconstruction of Unobserved States (PICRUSt) (20). Statistical analyses were conducted with STAMP, and functional differences in orthologs among groups were assessed by a one way ANOVA followed by Tukey-Kramer multiple comparisons (21).

### Culture of *Clostridium tyrobutyricum*

*Clostridium tyrobutyricum* (*C. tyroburyricum*, ATCC25755) was purchased from ATCC. *C. tyroburyricum* were cultured in supplemented medium for 8 h, and the culture broth was centrifuged at 5000 ×g for 10 mins. The *C. tyrobutyricum* were suspended in sterile PBS. The culture supernatant of *C. tyrobutyricum* was collected and sterilized with a 0.22μmfilter.

### SCFAs concentrations assay

SCFA analysis was performed as described (22). Small intestine contents samples and *C. tyrobutyricum* culture supernatant were thawed, processed using vacuum distillation, and analyzed using gas chromatography on an Agilent 7890A GC system with an HP-INNO WAX column (30 m × 0.32 mm × 0.5 μm) and flame ionization detector (Agilent Technologies Inc., CA, USA). Results were reported as mmol/g according to the original weight of contents used, excluding their moisture values, as SCFAs are contained within the solid phase of the contents. All measurements were performed in triplicate.

### Sodium butyrate and *Clostridium tyrobutyricum* administration experiment

Sodium butyrate (SB) (Sigma Aldrich) was >97% high-performance liquid chromatography purity. SB (200 mg/kg) or *C. tyrobutyricum* (10^8^ CFU/200 µL PBS) was orally administered to mice daily from -14 dpi to 7 dpi and mice (n =6 per group) were infected with 500 ML for establishing the acute infectious model. 6 mice per group were sacrificed using CO_2_ asphyxiation at 7 dpi. Small intestine and content were immediately collected for the following experiment. Intestinal adult worms were collected at 7 dpi. The remaining mice (n=6 per group) were sacrificed using CO_2_ asphyxiation and ML were recovered and counted at 35 dpi.

### Statistical analysis

All results were expressed as the mean ± SD. N refers to the number of mice used. The data shown are representative of three independent experiments. Statistical differences were determined using a 2-tailed Student’s t test or 1-way analysis of variance (Tukey honestly significant difference multiple comparison test) using GraphPad Prism (GraphPad Prism 8 Software, San Diego, CA). A P values are expressed as *p<0.05, **p< 0.01 and ***p<0.001.

## Results

### Gut microbiota played a role in LNT –triggered expulsion of *T. spiralis*

**Figure 1:**
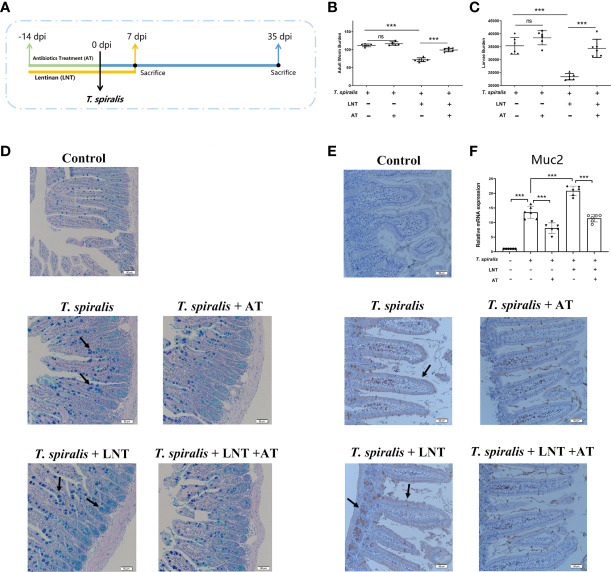
LNT failed to reduce the burden of *T. spiralis* in gut microbiota-dysbiosis mice. **(A)** Mice were administered an antibiotic treatment (AT) and LNT. **(B)** Adult worms (7 dpi) and **(C)** muscle larvae (35 dpi) were recovered from mice in each group, and the abundance of *T. spiralis* was calculated. **(D)** Periodic acid Schiff (PAS) staining (200×) of duodenal tissues (Scale bars: 50 μm). **(E)** Immunohistochemistry of duodenal tissues with anti-Muc2 antibody. **(F)** mRNA level of Muc2. Data are presented as the mean ± sd (n=6). The data shown are representative of three independent experiments. ns, not significant and ***p<0.001 as indicated by the line (one-way ANOVA with Tukey’s post test). These figures are representative of three independent experiments.

To evaluate the role of type 2 immunity in the effect of LNT on *T. spiralis* infection, mice infected with *T. spiralis* were administered a STAT6 inhibitor. We demonstrated that LNT significantly reduced the adult worm at 7 dpi and muscle larvae burden at 35 dpi. Interestingly, although inhibition of the IL-4 and IL-13 (type 2 cytokines) levels induced an increased burden of *T. spiralis*, we found that LNT still significantly reduced the burden of helminth (**Figures S1A, B**). Emerging evidence has revealed that the composition of intestinal microbiota is an important factor in regulating mucus barrier function in the intestine (7). To determine the role of gut microbiota in LNT –triggered host defence against *T. spiralis* infection, one group of mice administered an antibiotic treatment (AT) and LNT for comparison (**Figure 1A**). Adult worm and larvae burden were not significantly different in *T. spiralis* –infected mice with or without AT, however, the burden in mice with LNT + AT was significantly higher than the LNT alone group (**Figures 1B, C**), suggesting that the deworming capacity of LNT were impaired by AT. Moreover, mucus secretion was decreased in AT –induced gut microbiota-dysbiosis mice compared to the mice without AT. The expression of Muc2 induced by LNT was also significantly inhibited by AT (**Figures 1D–F**).

### LNT administration shifted the gut microbiota in *T. spiralis*-infected mice

**Figure 2:**
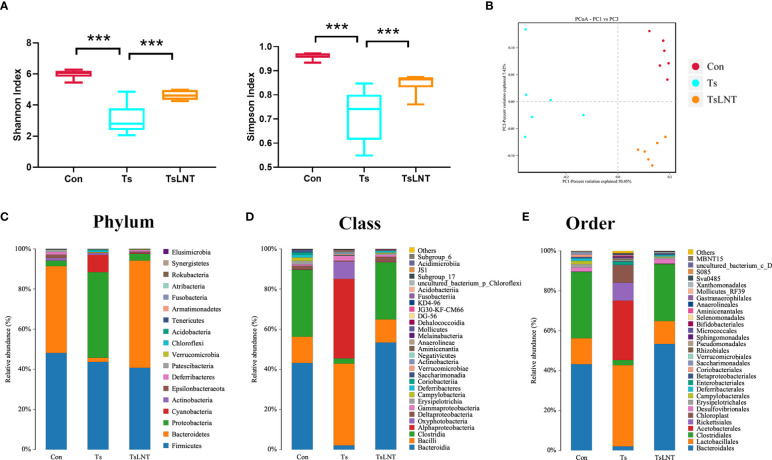
Characterization of the gut microbiota in mice after administration of LNT in *T. spiralis* –infected mice. The microbiota composition in the small intestine contents was analyzed by 16S rRNA gene sequencing (n =6). **(A)** Shannon index and Simpson index (α diversity). **(B)** Principal coordinates analysis (PcoA) plot (β diversity) ***p<0.001 as indicated by the line (one-way ANOVA with Tukey’s post test). **(C)** Composition of abundant bacteria at the phylum level. **(D)** Composition of abundant bacteria at the class level. **(E)** Composition of abundant bacteria at the order level.

**Figure 3:**
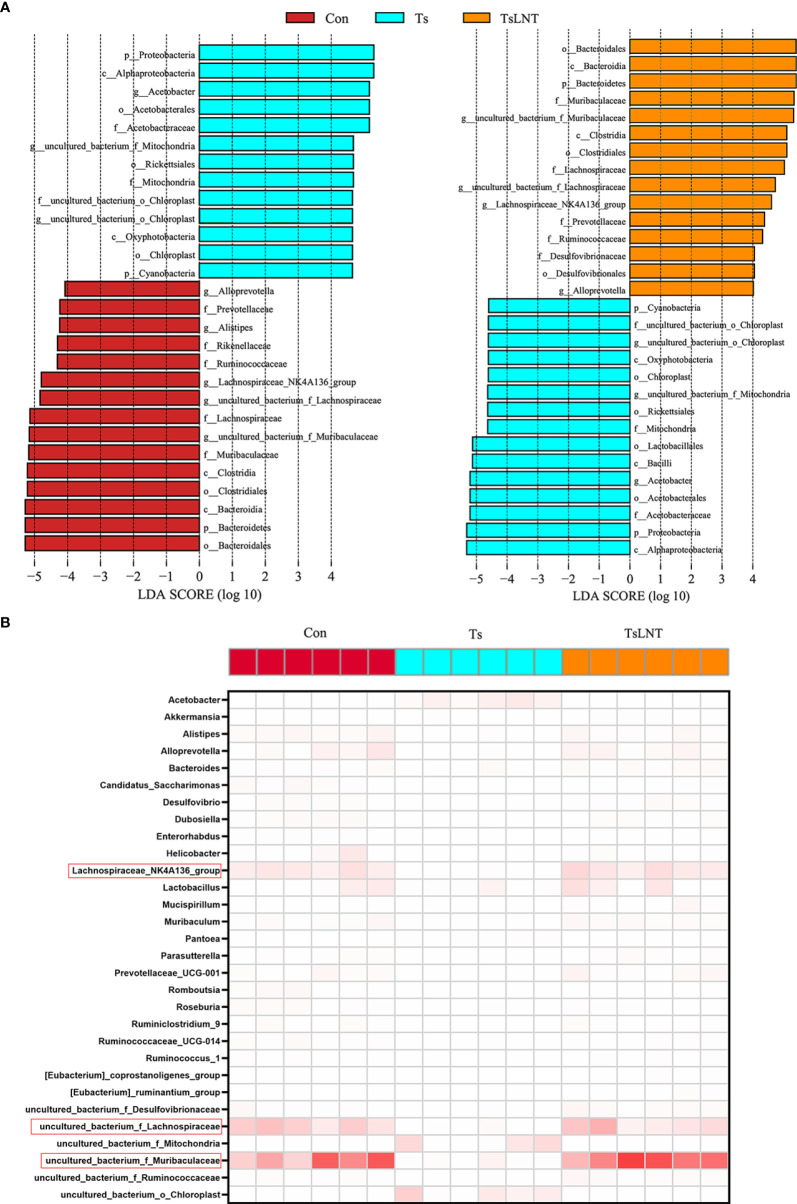
The most differentially significant abundant taxa enriched in microbiota by linear discriminant analysis (LDA). **(A)** Linear discriminant analysis (LDA) effect size showing the most differentially significant abundant taxa enriched in microbiota from the control group (Con), *T. spiralis* group (Ts), and *T. spiralis* + LNT group (TsLNT). **(B)** Heatmap of the abundance of bacteria at the genus level from the Con, Ts and TsLNT groups.

To investigate the effects of LNT on gut microbiota, 16S rRNA gene sequencing was performed. We showed that *T. spiralis* infection significantly decreased α-diversity in the Shannon index and Simpson index. The α-diversity was significantly increased in *T. spiralis* –infected mice with LNT administration (**Figure 2A**). A clear separation among the control group and *T. spiralis* group with or without LNT was found using UniFrac-based principal component analysis (PCoA) to evaluate the β-diversity (**Figure 2B**). *T. spiralis* infection significantly reduced the relative abundance of *Bacteroidetes* and enhanced *Proteobacteria* compared to controls (both p < 0.05), while LNT restored the abundance of *Clostridiales* and *Bacteroidales* to the control group level and significantly decreased the abundance of *Proteobacteria* compared to the *T. spiralis* group (**Figures 2C–E**). Linear discriminant analysis effect size (LEfSe) indicated that bacteria belonging to *Clostridia* and *Bacteroidia* were enriched in gut bacterial communities by LNT (LDA score >4) (**Figure 3**). Notably, the amount of *Muribaculaceae* and *Lachnospiraceae* NK4A136 in LNT-fed mice was restored (**Figure 3**).

In level two KEGG pathways, 22 functional orthologs were altered significantly in the Ts group compared with the control group (**Table 1**). LNT was related to the obvious changes in microbial function among these functional orthologs, including cell motility, cellular community prokaryotes, transport and catabolism, metabolism of other amino acids, infectious diseases: parasitic, glycan biosynthesis and metabolism. Importantly, the abundance of functional genes related to parasitic infectious diseases increased in the *T. spiralis* -infected mice, but LNT could significantly decrease the abundance of these functional genes. In addition, LNT increased the functional genes related to glycan biosynthesis and metabolism compared to *T. spiralis* -infected mice.

**Table 1:** Predicted KEGG functional pathway differences at level 2 inferred from 16S rRNA gene sequences using PICRUSt during T. spiralis infection with or without LNT.

Functional categories						
Level 1	Level 2	Con:mean ± sd(%)	Ts:mean ± sd(%)	TsLNT:mean ± sd(%)	Con vs Ts p value	TsLNT vs Ts p value
Cellular Processes	Cell motility	1.227± 0.251	0.173± 0.098	1.012± 0.271	<0.001	<0.001
	Cellular community - prokaryotes	1.468± 0.079	1.183± 0.115	1.472± 0.032	0.001	0.002
	Transport and catabolism	0.278± 0.07	0.138± 0.025	0.303± 0.038	0.005	<0.001
Environmental Information Processing	Signal transduction	2.772± 0.171	2.181± 0.127	2.691± 0.137	<0.001	<0.001
Genetic Information Processing	Folding, sorting and degradation	1.586± 0.03	1.727± 0.06	1.573± 0.022	0.002	0.001
Human Diseases	Cancers: Overview	0.507± 0.025	0.686± 0.035	0.501± 0.01	<0.001	<0.001
	Drug resistance: Antimicrobial	1.01± 0.025	0.862± 0.039	1.023± 0.03	<0.001	<0.001
	**Infectious diseases: Parasitic**	0.022± 0.005	0.046± 0.007	0.037± 0.002	<0.001	0.007
	Cancers: Specific types	0.046± 0.007	0.177± 0.046	0.045± 0.003	0.001	0.001
	Neurodegenerative diseases	0.132± 0.032	0.354± 0.166	0.114± 0.01	0.001	0.002
	Infectious diseases: Viral	0.006± 0.006	0.044± 0.029	0.005± 0.002	0.027	0.034
Metabolism	Metabolism of other amino acids	1.333± 0.056	1.781± 0.052	1.37± 0.081	0.029	0.029
	Metabolism of terpenoids and polyketides	1.07± 0.013	1.183± 0.017	1.054± 0.015	0.030	0.023
	Xenobiotics biodegradation and metabolism	0.757± 0.081	1.34± 0.144	0.821± 0.088	0.032	0.031
	Energy metabolism	3.852± 0.181	4.569± 0.189	3.839± 0.023	0.000	0.000
	Biosynthesis of other secondary metabolites	1.038± 0.06	0.727± 0.104	1.062± 0.015	<0.001	<0.001
	Glycan biosynthesis and metabolism	1.558± 0.193	1.169± 0.024	1.611± 0.093	<0.001	<0.001
	Carbohydrate metabolism	10.04± 0.266	9.078± 0.541	10.15± 0.177	<0.001	<0.001
Organismal Systems	Nervous system	0.207± 0.009	0.124± 0.025	0.215± 0.008	<0.001	0.001
	Aging	0.282± 0.016	0.407± 0.057	0.272± 0.001	0.006	<0.001
	Immune system	0.077± 0.005	0.044± 0.017	0.079± 0.003	0.009	0.005
	Circulatory system	0.004± 0.005	0.055± 0.04	0.001± 0.001	<0.001	<0.001

### LNT increased the concentration of butyrate in *T. spiralis* –infected mice

**Figure 4:**
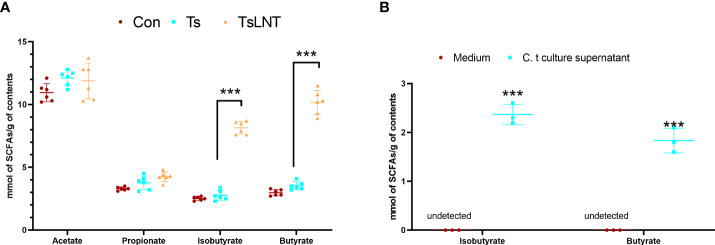
Concentrations of short-chain fatty acids (SCFAs). SCFA analysis of **(A)** small intestine contents samples (n=6) and **(B)** *C. tyrobutyricum* culture supernatant (n=3) was performed. Data are presented as the mean ± sd. The data shown are representative of three independent experiments. ***p<0.001 as indicated by the line (one-way ANOVA with Tukey’s post-test).

SCFAs are associated with mucus in the intestine (7). It has also been observed that SCFAs, such as acetate and butyrate, stimulate Muc2 expression in intestinal epithelial cells and increase mucus production and secretion (7). No statistically significant difference was observed in the concentrations of acetate and propionate between the Ts group and Ts+LNT group, whereas a significant decrease in the concentration of butyrate was noted (**Figure 4A**). In addition, the levels of butyrate in *C. tyrobutyricum* culture supernatant was shown in **Figure 4B**.

### Sodium butyrate and *Clostridium tyrobutyricum* triggered the expulsion of *T. spiralis*

**Figure 5:**
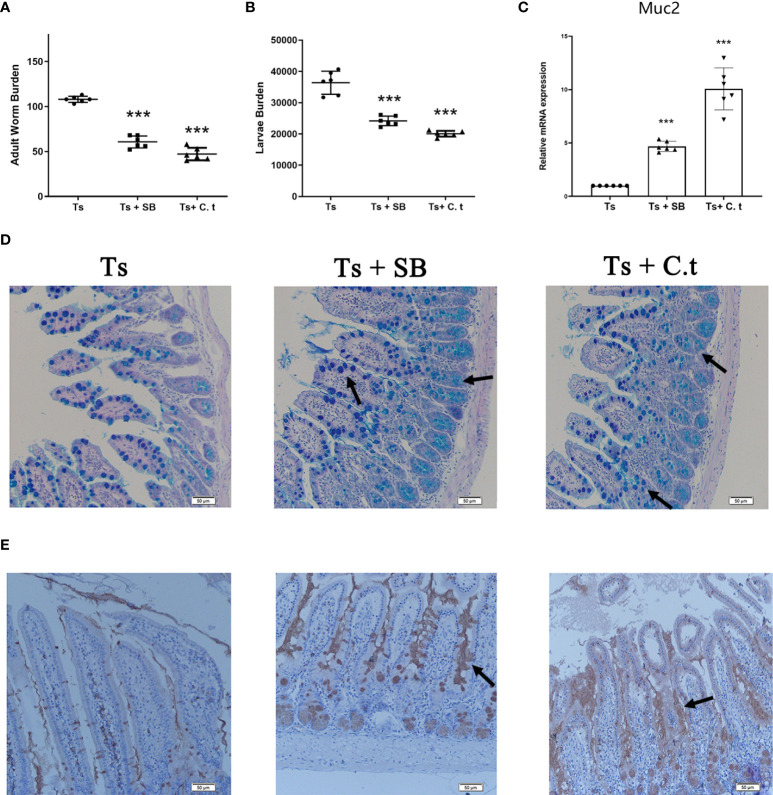
Sodium butyrate (SB) or *Clostridium tyrobutyricum* (C. t) reduced the burden of *T. spiralis* by promoting mucus production. SB (200 mg/kg) and C.t (10^8^ CFU/200 µL PBS) were orally administered to mice daily from -14 dpi to 7 dpi and mice (n =6 per group) were infected with 500 ML to establish the acute infectious model. Six mice per group were sacrificed using CO_2_ asphyxiation at 7 dpi. The remaining mice (n=6 per group) were sacrificed using CO_2_ asphyxiation and ML were recovered and counted at 35 dpi. **(A)** Adult worms (7 dpi) and **(B)** muscle larvae (35) dpi were recovered from mice in each group and the burden of *T.spiralis* was calculated. **(C)** mRNA level of Muc2. Data are presented as the mean ± sd (n=6). **(D)** Periodic acid Schiff (PAS) staining (200×) of duodenal tissues (Scale bars: 50 μm). **(E)** Immunohistochemistry of duodenal tissues with anti-Muc2 antibody. The data shown are representative of three independent experiments. ***p<0.001 as indicated by the line (Dunnett’s multiple comparison following ANOVA). These figures are representative of three independent experiments.

Butyrate is a primary product of gut microbial fermentation (23). C. *tyrobutyricum* is a probiotic, that is a butyrate-producing bacterium (24). To evaluate the effect of butyrate against *T. spiralis* infection, mice were orally administered with SB or *C. tyrobutyricum* daily. Both SB and *C. tyrobutyricum* significantly decreased the burden of adult worms at 7 dpi and muscle larvae at 35 dpi in mice (**Figures 5A, B**). Consistently, SB and *C. tyrobutyricum* increased the numbers of goblet cells and promoted the secretion of mucus and Muc2 expression (**Figure 5C–E**).

## Discussion

LNT has immunity-enhancing effects and we showed the adjuvanticity of LNT against helminth *T. spiralis* infection in the previous study (12). It has been reported that polysaccharides such as LNT can provide energy for gut microbiota in the intestine, and are associated with the homeostasis of the gut microbial community and overall host health (25). Intestinal dysbiosis participates in the pathogenesis of infections (26). In addition, LNT can ameliorate bacterial LPS –induced intestinal inflammation through gut microbiota (27). In this paper, we aimed to investigate the effect of oral LNT on helminth expulsion *in vivo*. We observed that an impaired type 2 immune response did not eliminate the capacity of worm expulsion by LNT; however, the gut microbiota contributed to LNT-triggered expulsion of helminth in the intestine by promoting the level of mucus. The intestinal mucus produced by goblet cells is a key player in the defense against enteric pathogens (2, 28). Increased goblet cell mucus secretion lead to alterations in the protective properties of the mucus barrier, which can directly or indirectly affect parasite establishment. It constitutes part of the “weep” response that develops to promote worm expulsion. Hyperplasia of goblet cells that produce mucin occurs in helminth infections, including *Trichinella spiralis*, *Trichuris muris*, and *Nippostrongylus brasiliensis* (29–31). Muc2 is the main source of mucin within the intestine and lack of Muc2 results in delayed expulsion of helminths in the intestine (32). Our results demonstrated that LNT significantly elevated the expression of Muc2, suggesting that LNT promotes mucus production, thereby driving the expulsion of intestinal helminths.

The microbiome plays a major role in mucus changes (7). We demonstrated that LNT administration ameliorated the shift in gut microbiota composition induced by *T. spiralis* infection. The results of 16S rRNA gene sequencing analysis demonstrated that *T. spiralis* infection decreased the diversity of gut microbiota composition whereas LNT restored the abundance of *Bacteroidales* and *Clostridiales* to the control group level. Notably, the *Muribaculaceae* and *Lachnospiraceae* families were enriched in the gut bacterial communities restored by LNT. *Muribaculaceae* family was named as S24-7 at first. One genera of this family may be essential for glycan degradation (33), indicating that mucus production by LNT is enhanced, leading to an enriched *Muribaculaceae* family. Furthermore, *Lachnospiraceae* are a family of anaerobic bacteria in the *Clostridiales* order within the *Firmicutes* phylum, including species previously identified as *Clostridium* clusters (34) and accumulate near the mucosa, allowing them to affect the host epithelial and mucosal immune system (35). The *Lachnospiraceae* can promote the colonization resistance of microorganisms to pathogens through metabolites (36). *Lachnospiraceae* NK4A136 group involves in metabolic disease (37). The *Lachnospiraceae* family also affects enterocytes by producing SCFAs such as butyrate (38). It is indeed well known that 16S rRNA sequencing allows genus-level resolution at most, which is a limitation of this study, while shotgun metagenomics sequencing will provide more meaningful functional annotation data, which is worth investigating in the future. Interestingly, we consistently demonstrated that LNT significantly enhanced the levels of butyrate, indicating that this metabolite may drive expulsion function of *T. spiralis* in the intestine.

Other studies have demonstrated that gavaging probiotics enables mice to regain faster intestinal SCFA production (39), such as butyrate (40) and SCFAs are absorbed and used by colonocytes to recover part of the energy spent in the intensive synthesis and secretion of mucus (41). Administration of butyrate promotes clearance of intestinal pathogen (*Citrobacter rodentium*) infection by enhancing mucus secretion (42). *C. tyrobutyricum* resides in the gut and has a protective role in defending against pathogens by regulating gut microbiota metabolites, such as butyrate (43). We evaluated the effect of butyrate and probiotics against helminth infection along with the effect on the level of mucus. Our results showed that butyrate and butyrate-producing bacteria could significantly decrease helminth burden and promote the production of mucus. Another study of ours found that barely β-Glucan-triggered *Akkermansia muciniphila* expansion facilitates the expulsion of intestinal helminth (10). Different sources of β-glucan can affect different probiotics to play a role in deworming. Therefore, targeted probiotics and related metabolite intervention may restore a normal microenvironment for the treatment or prevention of helminth infections.

**Figure 6:**
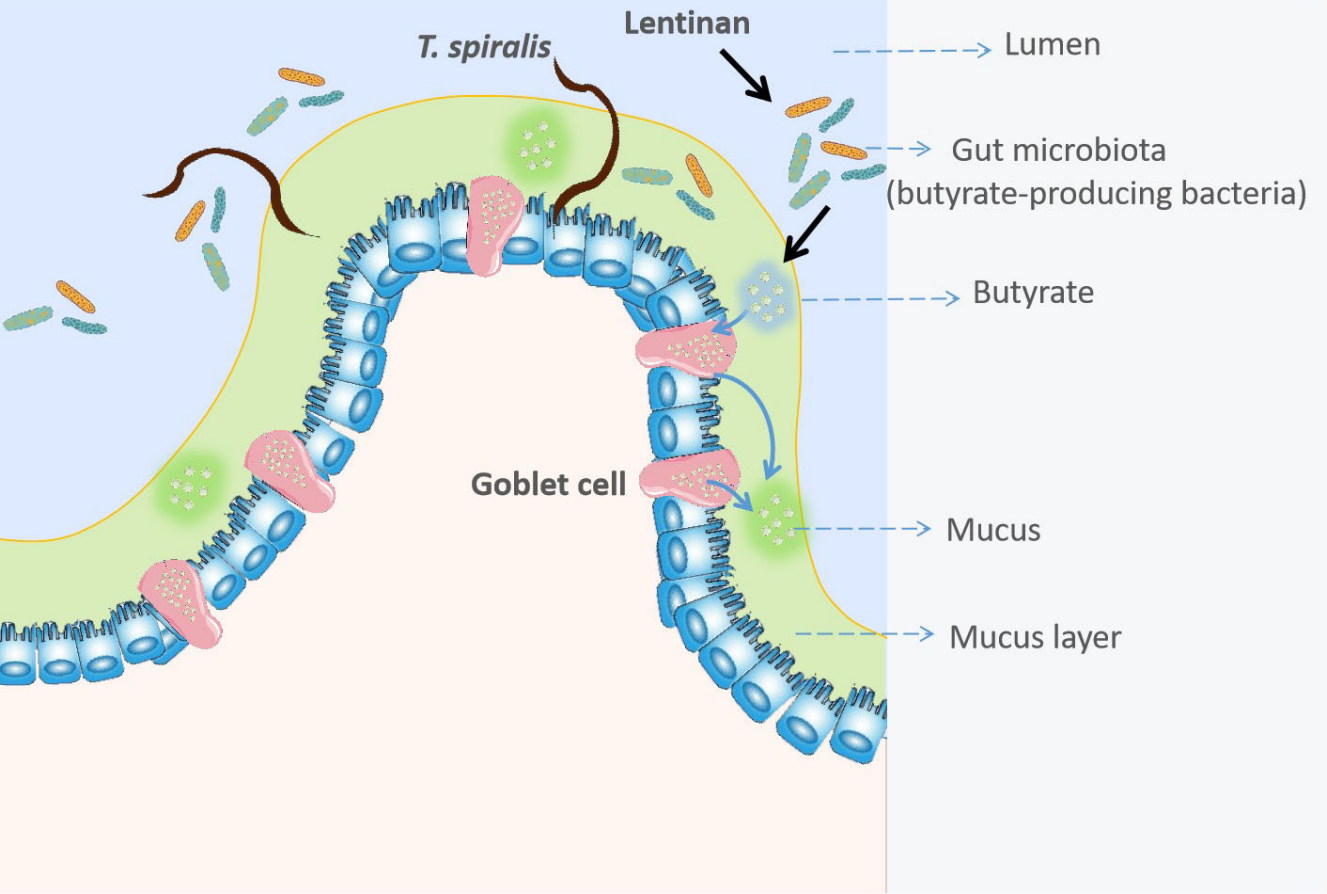
Lentinan -triggered butyrate-producing bacteria drive the expulsion of the intestinal helminth *Trichinella spirali*.

In conclusion, these results first revealed that LNT could promote mucus production and drive the expulsion of helminths. The butyrate-producing bacteria, *C. tyrobutyricum* also reduced the burden of helminths (**Figure 6**). Taken together, these findings of this study are easily implementable strategy to facilitate expulsion of gastrointestinal helminths.

## Data availability statement

The datasets presented in this study can be found in online repositories. The names of the repository/repositories and accession number(s) can be found below: NCBI, accession ID: PRJNA723732.

## Ethics statement

The animal study was reviewed and approved by Institutional Animal Care and Use Committee of Jilin University.

## Author contributions

XJ: Conceptualization, Writing- Original draft, Methodology, Software. YL.: Data curation, Writing- Original draft, Methodology. IV: Visualization, Investigation, Methodology. GK: Writing- Reviewing and Editing. ML: Funding acquisition, Writing- Reviewing and Editing, Supervision. XL: Funding acquisition, Writing- Reviewing and Editing, Conceptualization, Supervision. All authors contributed to the article and approved the submitted version.

## Funding

This study was supported by the National Key Research and Development Program of China (2021YFC2600202); the National Natural Science Foundation of China (31520103916, 31872467); Guangdong Innovative and Enterpreneurial Research Team Program (2014ZT05S123); Jilin Provincial Science and Technology Development Project (20180520042JH); and Program for JLU Science and Technology Innovative Research Team.

## Conflict of interest

The authors declare that the research was conducted in the absence of any commercial or financial relationships that could be construed as a potential conflict of interest.

## Publisher’s note

All claims expressed in this article are solely those of the authors and do not necessarily represent those of their affiliated organizations, or those of the publisher, the editors and the reviewers. Any product that may be evaluated in this article, or claim that may be made by its manufacturer, is not guaranteed or endorsed by the publisher.
