# Supplementary material for: Lentinan -triggered butyrate-producing bacteria drive the expulsion of the intestinal helminth Trichinella spiralis in mice
Source: Front Immunol. 2022 Jul 28;13:926765. doi: 10.3389/fimmu.2022.926765 (PMC9371446; doi:10.3389/fimmu.2022.926765)
Supplement: Supplementary file 1 [file Image_1.pdf]

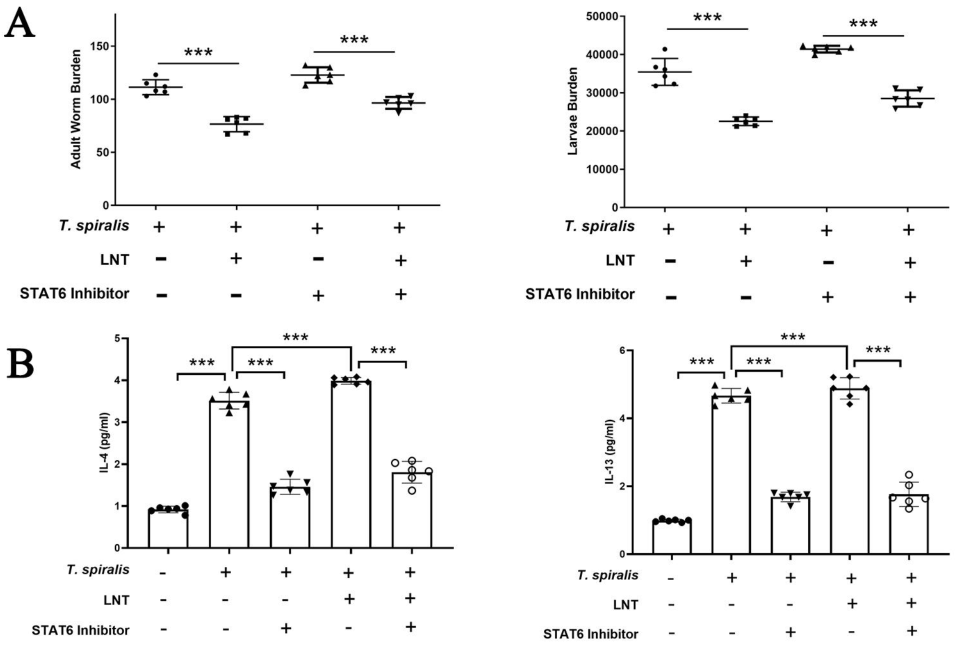

Fig. S1 Lentinan (LNT) could still reduce the burden of *T. spiralis* in the absence of type 2 immunity.

Mice were orally administered LNT (10 mg/kg) daily from -14 days post infection (dpi) to 7 dpi, the STAT6 inhibitor AS1517499 (20 mg/kg i.p.) at 1, 3, and 5 dpi and infected with 500 muscle larvae. (A) Adult worms (7 dpi) and muscle larvae（35) dpi were recovered from mice in each group, and the abundance of *T. spiralis* was calculated. (B)The levels of IL-4 and IL-13 in serum. Data are presented as the mean ± sd (n=6). The data shown are representative of three independent experiments. *p<0.05, **p< 0.01, and ***p<0.001 as indicated by the line (one-way ANOVA with Tukey's post test).
